# Role of VEGF-A and LRG1 in Abnormal Angiogenesis Associated With Diabetic Nephropathy

**DOI:** 10.3389/fphys.2020.01064

**Published:** 2020-08-31

**Authors:** Afei Zhang, Huawei Fang, Jie Chen, Leyu He, Youwei Chen

**Affiliations:** Department of Nephrology, The Second Affiliated Hospital of Jiaxing University, Jiaxing, China

**Keywords:** diabetic nephropathy, abnormal angiogenesis, VEGF-A, LRG1, anti-angiogenesis

## Abstract

Diabetic nephropathy (DN) is an important public health concern of increasing proportions and the leading cause of end-stage renal disease (ESRD) in diabetic patients. It is one of the most common long-term microvascular complications of diabetes mellitus that is characterized by proteinuria and glomerular structural changes. Angiogenesis has long been considered to contribute to the pathogenesis of DN, whereas the molecular mechanisms of which are barely known. Angiogenic factors associated with angiogenesis are the major candidates to explain the microvascular and pathologic finds of DN. Vascular endothelial growth factor A (VEGF-A), leucine-rich α-2-glycoprotein 1, angiopoietins and vasohibin family signal between the podocytes, endothelium, and mesangium have important roles in the maintenance of renal functions. An appropriate amount of VEGF-A is beneficial to maintaining glomerular structure, while excessive VEGF-A can lead to abnormal angiogenesis. LRG1 is a novel pro-angiogenic factors involved in the abnormal angiogenesis and renal fibrosis in DN. The imbalance of Ang1/Ang2 ratio has a role in leading to glomerular disease. Vasohibin-2 is recently shown to be in diabetes-induced glomerular alterations. This review will focus on current understanding of these angiogenic factors in angiogenesis and pathogenesis associated with the development of DN, with the aim of evaluating the potential of anti-angiogenesis therapy in patients with DN.

## Introduction

Diabetic nephropathy (DN) is the most common cause of end-stage renal disease (ESRD) in developed countries (Fu et al., 2018). According to the data of International Diabetes Federation, the global diabetes prevalence is estimated to be 463,000,000 people in 2019, and it will rise to 578,000,000 and 700,000,000 by 2030 and 2045, respectively, about 30–40% of whom will develop DN (Saeedi et al., 2019). Currently, therapies for DN include intensive blood glucose control, as well as blood pressure control by inhibition of renin–angiotensin–aldosterone system (Kato and Natarajan, 2019). However, these therapeutic strategies only provide limited renal protective effects in preventing DN progressing to ESRD. It is therefore necessary to understand the molecular mechanisms related to DN in-depth.

Angiogenesis is a complex physiological process that involves the interaction between many angiogenic growth factors and endothelium and extracellular matrix (Ucuzian et al., 2010). It requires endothelial cell (EC) proliferation, survival, migration, morphology changes, anastomoses, and extracellular matrix degeneration (Negri et al., 2020). Angiogenesis is mediated by the delicate balance of stimulatory and inhibitory growth factors, while any disruption of these factors would give rise to abnormal angiogenesis with pathological outcomes. Many studies have observed abnormal blood vessels around glomeruli of diabetic mouse, which may contribute to the glomerular dysfunctions in DN (Nyengaard and Rasch, 1993; Guo et al., 2005). Therefore, angiogenic growth factors involved in angiogenesis may be potential therapeutic targets for DN. The previous therapy was mainly focused on vascular endothelial growth factor A (VEGF-A). The VEGF-A inhibitors have previously shown some beneficial effects on diabetic ocular disease and DN experimental mice models, but recent evidences raised controversial opinions on its use in clinical practice. Several lines of evidence revealed that patients accepted anti-VEGF treatment developed worsening proteinuria and glomerular microangiopathy (Hanna et al., 2019; Touzani et al., 2019), while other studies supported that it is unlikely to cause any renal injury (Kameda et al., 2018; O’Neill et al., 2019). Leucine-richα-2-glycoprotein 1 (LRG1) is a novel pro-angiogenic factor that has demonstrated to promote angiogenesis by enhancing ALK-1signaling pathway in both diabetic ocular disease and DN mouse models (Wang et al., 2013; Haku et al., 2018; Hong et al., 2019). The imbalance of Ang1/Ang2 ratio has a role in leading to glomerular disease (Loewen et al., 2019). Vasohibin-2 is recently shown to be in diabetes-induced glomerular alterations (Masuda et al., 2018). In this review, we summarize how these angiogenic factors mediate abnormal angiogenesis associated with DN, with the aim of evaluating their potential in developing innovative therapeutic strategies.

## Abnormal Angiogenesis and DN

As a highly vascularized organ, the kidney vascular system receives approximately 20% cardiac output, it plays an important role in controlling various homeostasis functions of the kidney such as waste removal, acid-base balance, liquid electrolyte homeostasis, and oxygen delivery (Sequeira Lopez, 2016). Therefore, ensuring the normal blood vessel formation in kidney is of great importance for maintaining renal function. However, conditions such as high glucose, high blood pressure, hypoxia, and oxidative stress usually lead to pathologic angiogenesis in diabetes (Kota et al., 2012).

The microangiopathy in diabetic kidney includes the excessive and abnormal angiogenesis in early stage of DN, and the rarefaction of capillaries in the glomeruli in advanced stage of DN (Fadini et al., 2019). The aberrant and excessive angiogenesis in early stage of DN is structurally immature and high permeability that would contribute to plasma protein extravasation (Nakagawa et al., 2009; Tanabe et al., 2017). In addition, the excessive vessels result in an enlarged glomerular filtration surface, which induces a harmful glomerular filtration rate that is significantly above normal levels in early stage of DN (Nyengaard and Rasch, 1993). Which may lead to severer glomerular dysfunction such as rapid progression of glomerular filtration rate decline and increased albuminuria. While the capillary rarefaction would decrease supplement of blood and oxygen, this leads to the loss of tubular cell viability, interstitial and glomerulosclerosis fibrosis (Afsar et al., 2018). Recently, Filinova et al. (2019) studied the pathomorphological features of STZ-induced diabetic mice model in the development of DN by using morphometrics method. Their findings revealed that the diabetic kidney is characterized by increased glomerular volume and significant glomerular capillary hyperemia. Moreover, the increased mesangial cell proliferation and thickened capillary basement membrane were observed as well. Finally, the urinary albumin excretion increased by 1.6 times (Filinova et al., 2019). Moreover, the observed results supported the fact that there exists abnormal angiogenesis in glomeruli of DN mice model, which is in accordance with previous study (Osterby and Nyberg, 1987). It is reported the microangiopathy in diabetic patients results in thickening of the glomerular capillary basement membrane and the expansion of the mesangial matrix (Tsilibary, 2003). Liu et al. (2018) reported that the Slit2 derived from mesangial cells is observed to promote capillary-like-networks in human renal glomerular endothelial cell via Slit2/Robo1 signaling pathway under diabetic-like environment. Further study revealed that Slit2 promotes angiogenesis by increasing the expression of VEGF-A (Liu et al., 2018). It is reported that the thickened GBM is always accompanied with increased capillary number and area in early stage of DN (Diani et al., 1987; Nyumura et al., 2012; Marshall, 2016). Mesangial cell is of great importance for maintaining the dynamic balance of the mesangial matrix and structural integrity of the glomerular capillary tuft (Nguyen and Goldschmeding, 2019). Renal biopsy analysis revealed that the herniation of expanded mesangium starts with deposition of worn-out GBM material. This process is associated with the proliferation and outgrowth of glomerular vessels (Loewen et al., 2019). The findings demonstrated that the majority of the abnormal vessels consist of a continuous endothelium sitting on a thickened basement membrane surrounded by one or even two layers of smooth muscle cells. These vessels are easily free from the widened glomerulus entrance and are drained into peritubular capillaries, resulting in plasma exudations. Though the exact role of abnormal angiogenesis in glomerular alterations is not well understood, various studies have demonstrated that inhibition of angiogenesis can delay progression of the above glomerular alterations (Haraguchi et al., 1997; Yamamoto et al., 2004; Ichinose et al., 2005, 2006; Liu et al., 2018; Masuda et al., 2018). Taken together, abnormal angiogenesis may contribute, either directly or indirectly, to glomerular hypertrophy, GBM thickening, and mesangial expansion, which will benefit the progression of DN. Therefore, it is essential to further discuss the factors related to angiogenesis in DN.

## The Role of VEGF-A in Abnormal Angiogenesis of DN

### The Biological Characteristics of VEGF-A

VEGF is a specific growth factor for EC growth and differentiation, which is a critical mediator of vascular permeability and angiogenesis (Byrne et al., 2005). It is a 34–42-kDa homodimeric glycoprotein that has different binding affinities for the three receptor tyrosine kinases, which are VEGF receptor 1 (VEGFR1), VEGFR2, and VEGFR3 (Karayiannakis et al., 2003; Bhisitkul, 2006). The VEGF gene family contains VEGF-A, VEGF-B, VEGF-C, VEGF-D, and placental growth factor (PIGF) (Bhisitkul, 2006). Of the VEGF-family members, VEGF-A is the most studied factor and closely associated with angiogenesis, which is mainly mediated by VEGFR1 and VEGFR2 signaling pathway (Ollero and Sahali, 2015; Mahecha and Wang, 2017). It is organized into eight exons and separated by seven introns. There are at least nine isoforms of VEGF-A by alternative splicing of these exons, which are termed according to their amino-acid number, including VEGF 165, VEGF 121, VEGF189, and so on. All of them include exons 1–5 and the terminal exon (exon 8), except exons 6 and 7 that encode heparin-binding domains that can be included or excluded (Varey et al., 2008). The shorter isoforms, VEGF 121, lack both exons 6 and 7, while the longer isoforms, VEGF 145, VEGF189, and VEGF206, include both exons 6 and 7 (Peach et al., 2018). VEGF 165 is the predominant isoform that secreted approximately 46 kDa homodimer, which has 15 basic amino acids within the 44 residues encoded by exon 7 (Takahashi and Shibuya, 2005). In addition, exon 8 contains two 3′ splice site in its nucleotide sequences, which generates two types of isoforms. It generates VEGF xxx by using the proximal splice site (inclusion of exon 8a), a proangiogenic family of isoforms. However, when it splices out of exon 8a (inclusion of exon 8b), it would generate the antiangiogenic family of isoforms, the VEGF xxxb. For example, VEGF 165b is the first of these exon 8b-encoded isoforms, which can combine with VEGFR2 but poorly activates the VEGFR2 kinase domain (Varey et al., 2008).

**FIGURE 1:**
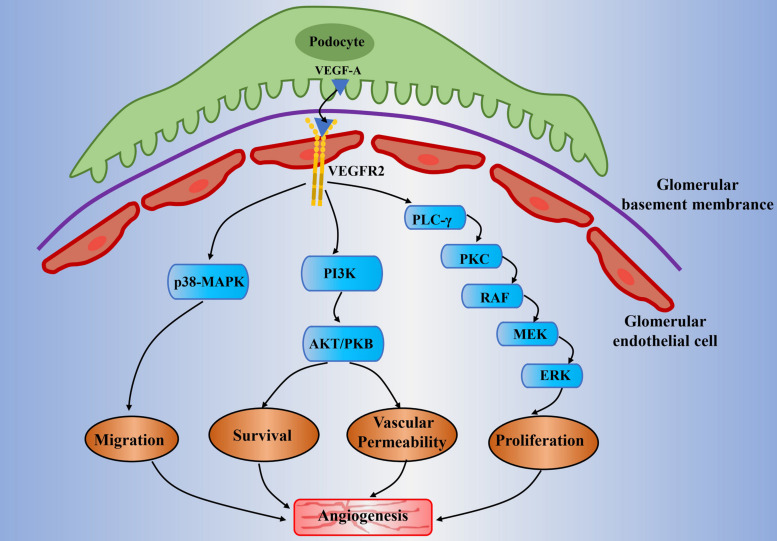
Potential mechanisms of abnormal angiogenesis mediated by VEGF-A/VEGFR2 signaling in diabetic nephropathy. The VEGF-A is mainly expressed in the podocytes. It will pass through the glomerular filtration barrier and will bind to VEGFR2 that is expressed on the glomerular endothelial cell. It subsequently activates the downstream signaling pathways which include PLC-γ/PKC, PI3K, and p38-MAPK. It is generally involved in angiogenesis through the following mechanisms: proliferation of endothelial cell (mitogen effects), increase of vascular permeability, migration of endothelial cell, and enhancement of signal transmission related to endothelial survival. Moreover, the high glucose condition would result in the upregulation of VEGF-A and decline of NO bioavailability (known as uncoupling of VEGF-A and NO). This will amplify the VEGF-A/VEGFR2 signaling pathways and will lead to abnormal angiogenesis and glomerular endothelial cells dysfunction in diabetic nephropathy. VEGF(R), vascular endothelial growth factor (receptor); PLC-γ, phospholipase-Cγ; PKC, protein kinase C; MEK, mitogen-activated protein kinase/extracellular signal-regulated kinase kinase; ERK, extracellular signal-regulated kinase; PI3K, phosphoinositide-3-kinase; AKT/PKB, protein kinase B; MAPK, mitogen-activated protein kinase; NO, nitric oxide.

The endothelial cellular responses to VEGF-A are primarily driven by the binding of VEGF-A and its receptors (Peach et al., 2018). It leads to physiological and pathological angiogenesis via promoting EC proliferation, EC migration, and tube formation (Niu and Chen, 2010; Simons et al., 2016; Moccia et al., 2019). VEGFR2 is the main mediator in promoting mitogenic, angiogenic, and vascular permeability effects (Ferrara et al., 2003; Shibuya, 2006) (Figure 1). The VEGF-A binding sites are initially located on the vascular ECs cell surface, the sites where VEGFR2 is expressed (Ferrara et al., 2003; Liu Z. Y. et al., 2017). The binding between VEGF-A and VEGFR2 undergoes dimerization and tyrosine phosphorylation and then activates the downstream signaling pathways in relation to angiogenesis in ECs and some cellular responses such as mitogenic and survival signal as well (Abhinand et al., 2016). Simultaneously, it induces the activation of several signaling proteins in ECs, which include phosphoinositide 3-kinase (PI3K), p38 mitogen-activated protein kinases (p38MAPK), and extracellular-signal-regulated kinases (ERK) (Ferrara et al., 2003; Koch et al., 2011; Abhinand et al., 2016). It can therefore promote angiogenesis by enhancing proliferation, survival, migration of ECs, and increasing permeability of existing vessels to form a lattice network for facilitating ECs migration (Niu and Chen, 2010). Unlike other VEGF-A isoforms, VEGF-A165b shows incomplete receptor phosphorylation and inactivation of the downstream signaling pathways relevant to angiogenesis (Woolard et al., 2004). In fact, it decreases ECs proliferation and migration result from VEGF-A and acts as an endogenous inhibitor of VEGF-A.

There are controversial opinions on the precise function of VEGFR1 on VEGF-A. Park et al. (1993) proposed that VEGFR1 negatively regulates the VEGF-A signaling pathway by preventing VEGF-A binding to VEGFR2 (Park et al., 1993). This suggests that it may not primarily be a receptor that produces mitogenic signal just as VEGFR2 does. In addition, a soluble form of VEGFR1 has been proved to inhibit VEGF-A activity (Kendall and Thomas, 1993). On the contrary, the other study has reported that VEGFR1 can produce mitogenic signals by interacting with various signal-transducing proteins under certain circumstances (Maru et al., 1998). Generally, VEGFR1 negatively regulates VEGF-A and VEGFR2 signaling pathway to provide the proper balance for a net positive effect on blood vessel formation and cell migration. But in diabetic kidney, the renal expression of VEGFR1 is decreased by 40%; this leads to the enhancement of VEGF-A and VEGFR2 signaling (Yang et al., 2014). On the other hand, VEGFR1 has been shown to be a positive mediator of VEGF-A and VEGFR2 signaling in the experimental models of some primary tumors and wet age-related macular degeneration (Huang et al., 2011). The potential mechanism may be due to the VEGFR1 that can negatively control the amount of VEGF-A signal that is sensed by endothelial cells, which lead to a compensatory increase in VEGF-A expression (Kearney et al., 2004).

### VEGF-A and Abnormal Angiogenesis of DN

The kidney eliminates metabolites, toxins, and electrolytes from the human body through a glomerular filtration barrier consist of a fenestrated endothelium, basement membrane, and podocytes (Bartlett et al., 2016; Fissell and Miner, 2018). Proteinuria caused by glomerular filtration barrier structure changes is the hallmark of DN, which include reduced endothelial fenestrations, thickened GBM, and podocyte loss (Satchell, 2012). VEGF-A is mainly expressed in glomerular podocytes and tubular cells, while the VEGFR2 is expressed predominantly in glomerular endothelial cells (Majumder and Advani, 2017). A previous study on experimental diabetic mice has observed the upregulation of VEGF-A and VEGFR2 in the kidney of an early stage DN (Cooper et al., 1999). It has demonstrated that there exists a paracrine VEGF-A and VEGFR-2 signaling pathway between podocytes and glomerular ECs (Cooper et al., 1999). The subsequent research revealed that the main signaling molecules include PI3K/Akt and ERK, which relate to nitric oxide (NO) production, glomerular ECs proliferation and migration (Nakagawa et al., 2006; Nakagawa, 2008; Veron et al., 2011). Specifically, the VEGF-A secreted by podocytes would cross contrary to urinary flow to bind with the VEGFR2 that expressed on the surface of glomerular ECs (Sison et al., 2010) (Figure 1). Veron et al. (2010) developed a transgenic mouse model that is controlled by the specific promoter for the glomerular podocyte protein (Podcin, NPHS2), which can induce the VEGF-A overexpression by using tetracycline. This mice model was shown to result in proteinuria, glomerular hypertrophy, GBM thickening, and mesangial expansion under non-diabetic milieu (Veron et al., 2010). It is worth noting that both pharmacological and endogenous VEGF-A inhibitors have exhibited renal protective effects in a variety of diabetic mice models, such as STZ-induced diabetic rats (De Vriese et al., 2001; Jin et al., 2012; Stevens et al., 2018). VEGF-A inhibitor sFlt-1 (soluble fms-related tyrosine kinase 1) is observed to reverse renal impairment by decreasing albuminuria, glomerular hypertrophy, and mesangial matrix expansion in mice diabetic mice. In addition, as reflected by decreased levels of vascular cell adhesion molecule 1 (VCAM-1), it also reduced VEGF-A-induced endothelial activation (Bus et al., 2017).

It is recently reported that because the VEGFR2 was blocked by using a VEGFR2 kinase inhibitor SU5416 in experimental diabetic mode, these mice models developed histological characteristics mimicking the key features of advanced human DN. They demonstrated that the blockade of VEGFR2 attenuates mesangial matrix expansion and basement membrane thickening, tubulointerstitial inflammation, and tubular atrophy. In addition, it also ameliorates the albuminuria in experimental type 2 diabetes (Lavoz et al., 2020). Evidenced by several latest studies, these renal protective effects may be due to the improved downstream signaling of VEGF-A and VEGFR2 related to angiogenesis. For example, the MiR-20a has been observed to ameliorate diabetic angiopathy by downregulating the protein expression of MAPK and ERK in STZ-induced DN mice models (Li et al., 2020). The overexpression of soluble neurite outgrowth inhibitor-B improves DN progression by preventing the impairment of tube formation via attenuating the phosphorylation of Akt, which relates to proliferation of ECs (Hernandez-Diaz et al., 2019).

Many studies have reported the reduced NO bioavailability in the diabetic kidney due to hyperglycemia, advanced glycation end-products, uric acid, and oxidative stress (Bucala et al., 1991; Brodsky et al., 2001; Ceriello et al., 2001; Khosla et al., 2005). On the other hand, the expression of VEGF-A is significantly upregulated under the diabetic-like environment. This can be described as VEGF-A and NO axis uncoupling, which has long been implicated in abnormal angiogenesis in DN (Nakagawa et al., 2006). Previous studies have shown excessive small vessels around glomeruli in mouse models of nitric oxide synthase knockout and NO blockade, and these experimental models displayed glomerular dysfunction of early stage in DN (Nakagawa et al., 2006, 2007; Veron et al., 2014). The uncoupling of VEGF-A with NO can enhance glomerular ECs proliferation and migration via activation of the ERK pathway (Nakagawa et al., 2006; Nakagawa, 2008). On the contrary, ECs proliferation in response to VEGF-A can be prevented by using an NO donor (Nakagawa et al., 2006). Furthermore, recent studies have demonstrated that partial reversion of uncoupling of VEGF-A with NO can improve endothelial dysfunction induced by high glucose in type 2 diabetic rats (Hou et al., 2014; Zhou et al., 2018). In total, current data suggests that the reduced NO bioavailability in diabetic kidney can further enhance VEGF-A/VEGFR2 signaling and subsequently induce more severely aberrant angiogenesis.

Several studies have demonstrated that the level of VEGF-A is increased in both kidney and urinary in the early stage of DN patients (Kanesaki et al., 2005; Kim et al., 2005; Hanefeld et al., 2016). Correspondingly, extra vessels around glomeruli and increased endothelial number are observed in diabetic human renal (Kanesaki et al., 2005; Hohenstein et al., 2006). Based on the above findings, the inhibition of abnormal angiogenesis via blocking VEGF-A signaling may be a possible therapy for DN patients. Previous studies have shown the beneficial effects of anti-VEGF-A in attenuating albuminuria, glomerular hypertrophy, and glomerular dysfunction in diabetic mice models (De Vriese et al., 2001; Flyvbjerg et al., 2002; Sung et al., 2006). To our knowledge, however, there is no anti-VEGF-A therapy for DN patients at present. In addition, the existed clinical use of VEGF-A inhibitors in diabetic retinopathy patients have shown controversial outcomes. As shown by electron microscopy evidence, patients who received intravitreal injection of bevacizumab are found to exhibit exceptional case of kidney injury related to glomerular microangiopathy, which includes the thickening of capillary wall and glomerular basement membrane (Touzani et al., 2019). In another study, diabetic patients accepted intravitreal anti-VEGF agents rapidly developed worsening proteinuria and renal function decline (Hanna et al., 2019). On the contrary, a current study showed that the average eGFR did not change in a total of 69 diabetic patients who received intravitreal anti-VEGF agents (Kameda et al., 2018). In addition, no episode of acute kidney injury occurred. Furthermore, the study by O’Neill et al. (2019) showed that long-term intravitreal anti-VEGF agents would not appear to increase the risk of developing or worsening proteinuria in patients with and without diabetic kidney disease. Such controversial results may be due to VEGF-A that has a crucial role in both maintaining glomerular vascular structure and repairing glomerular endothelial injuries (Shimizu et al., 2004). Therefore, therapies involving anti-VEGF-A in DN must keep a normal level. Otherwise, overinhibition of VEGF-A may lead to harmful side effects. Evidenced by a recent study, receiving intravitreal bevacizumab leads to worsening proteinuria and renal function in patients with early stage of DN, but it is subsequently improved by switching to a lower potency antibody, ranibizumab (Hanna et al., 2020).

## The Role of LRG1 in Abnormal Angiogenesis of DN

### The Biological Characteristics of LRG1

LRG1 is a highly conservative member of leucine-rich repeat family that firstly identified in human serum in 1977 (Haupt and Baudner, 1977). It is a secreted glycoprotein that mediates protein–protein interactions, which is widely involved in signal transduction, cell proliferation, cell migration, cell invasion, cell adhesion, cell survival, and cell apoptosis (Buchanan and Gay, 1996; Wang et al., 2013; Wang et al., 2015; Zhong et al., 2015; Zhang et al., 2016; Ban et al., 2019). The previous studies have revealed that LRG1 level is elevated in serum and urine of a variety of diseases, including inflammatory diseases (Fujimoto et al., 2015), ocular disease (Wang et al., 2013; Chen et al., 2019), and cancers (Kawakami et al., 2005; Zhou et al., 2017). Recently, LRG1 is identified as a novel pro-angiogenic factor that contributes to tumor-growth and diabetic retinopathy (Wang et al., 2013; Zhang et al., 2016). Wang et al. (2013) pointed out that LRG1 regulates the pathogenic angiogenesis in retinal disease mouse model in the presence of TGF-β1.

**FIGURE 2:**
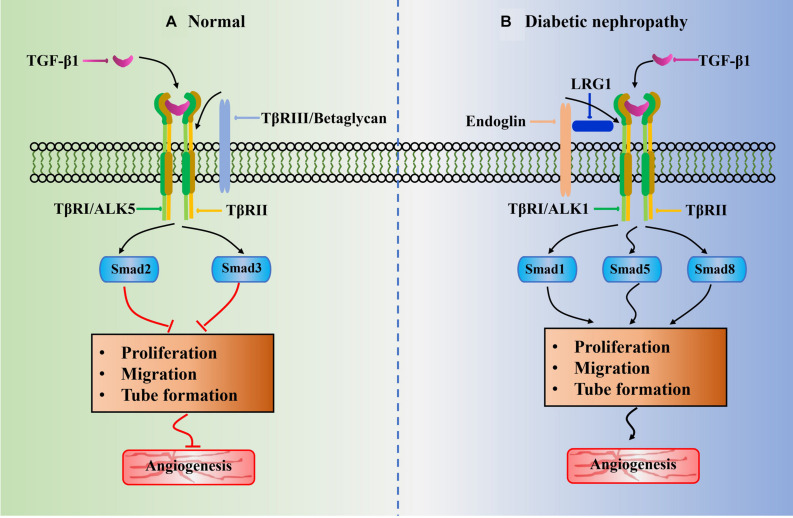
Schematic of potential role of LRG1 in abnormal angiogenesis of diabetic nephropathy associated with TGF-β1 signaling pathway. The black arrowhead lines represent positive crosstalk interactions and process and red flat-ended lines indicate inhibitory effects. **(A)** ALK5 receptor is widely existed in normal glomerular endothelial cell. The phosphorylation of Smad2/3 resulted from TGF-β1/ALK5 signaling would block angiogenesis by inhibition of EC proliferation, EC migration, and tube formation. **(B)** Under diabetic-like environment, high glucose leads to upregulation of LRG1. The LRG1 directly binds to endoglin and subsequently increases activation of the ALK1-mediated Smad1/5/8 pathway. It thus induces abnormal angiogenesis by promoting EC proliferation, EC migration, and tube formation. This is beneficial for the development of diabetic nephropathy.

TGF-β1 plays an important role in blood vessel formation with a highly context-dependent process, which is affected by ligand bioavailability and concentration, and receptor availability and internalization. It can switch from the principally anti-angiogenic functions to pro-angiogenic effects (Pardali et al., 2010; Massague, 2012). It exerts angiogenic biological effects by interacting with its specific TGF-β type I receptor (TβRI) and TGF-β type II receptor (TβRII) to induce the activation of TGF-β1/ALK1-mediated pathways (Goumans et al., 2009). Generally, the receptor phosphorylation activation is initiated by the TGF-β1 ligand binding to TβRII and followed by incorporation of TβRI to form a large ligand–receptor complex (Kubiczkova et al., 2012). It subsequently signals via two different type I receptors, namely, activin receptor-like kinase 1 (ALK1) and ALK5 (Franzen et al., 1993). Herein the ALK5 activation results in the Smad2/3 signaling to inhibit ECs proliferation, migration, and tube formation, leading to the anti-angiogenic functions (Figure 2A); whereas the ALK1 activation results in the Smad1/5/8 signaling to promote ECs proliferation, migration, and tube formation, leading to the pro-angiogenic effects (Figure 2B) (Goumans et al., 2002). In addition, beside the receptors TβRI and TβRII, betaglycan (also called TβRIII) and endoglin are also receptors of TGF-β1. Endoglin is an accessory receptor that is indirectly involved in TGF-β signaling via its crucial role in balancing ALK1 and ALK5 pathways (ten Dijke et al., 2008). It is highly expressed on proliferating vascular ECs and binds TGF-β1 ligands in association with TβRII. It is essential for efficient TGF-β1/ALK1 pathway but indirectly blocks TGF-β1/ALK5 pathway (Lebrin et al., 2004). Interestingly, a recent study revealed that the increased endoglin upregulated expression of VEGF-A in type 1 diabetic mouse model, and it is demonstrated to enhance glomerular ECs activation by regulating Akt signaling (Bus et al., 2018).

Notably, LRG1 is reported to interact with multiple types of TGF-β receptors, especially endoglin (Song and Wang, 2015). In a study related to retinal disease mice model, LRG1 is found to interact with endoglin and promote pathogenic angiogenesis via promoting the TGF-β1/ALK1 signaling pathway (Wang et al., 2013). Though LRG1 can associate with both ALK1 and ALK5, it is revealed that endoglin augments the interaction between LRG1 and ALK1, and the incorporation of TGF-β1 would further enhance this signaling (Lebrin et al., 2004; Wang et al., 2013). In addition, they also verified lesion sizes of choroidal neovascularization can be attenuated by using a series of LRG1 antibodies (Wang et al., 2013). It is therefore demonstrated that LRG1 plays an important role in angiogenesis. Lately, Zhang et al. (2019) further confirmed the role of LRG1 in pathological contexts. They incubated human umbilical vein endothelial cells (HUVECs) with exogenous LRG1. The migration and tube formation ability of HUVECs are observed to be enhanced significantly after addition LRG1, whereas the load-induced hypertrophic scar formation resulted from pathological angiogenesis is ameliorated after depletion of LRG1 (Zhang et al., 2019).

### TGF-β and DN

Many aspects of the diabetic state stimulate renal TGF-β activity, including hyperglycemia, increased glycated albumin and production of vasoactive agents such as angiotensin II (Kolm-Litty et al., 1998; Chen et al., 2001). The activated TGF-β induces extracellular matrix accumulation by stimulating mRNA expression and collagen production on one hand (Riser et al., 1996; Lee, 2012). On the other hand, it can also damage the extracellular matrix degradation by blocking the production of enzyme that degrade matrix (Ohno et al., 1995). In addition, it disturbs the normal regulation of cell cycle by inducing cyclin-dependent kinase inhibitors (e.g., p27Kip1 and p21) (Heino et al., 1989), which will result in renal cellular hypertrophy. Moreover, anti-TGF-β antibody treatment had been proven to reduce mesangial matrix expansion by preventing increased renal expression of matrix genes increases in the renal expression of matrix genes but it fails to attenuate the degree of albuminuria (Ziyadeh et al., 2000). This may be due to the increased albumin permeability across the GBM, which is affected by hemodynamic stress, increased membrane pore size, anionic charge reduction, and increased VEGF-A expression in DN (Ziyadeh et al., 2000).

Renal fibrosis is characterized by excessive extracellular matrix accumulation. The TGF-β is reported to promote renal fibrosis via Smad3 signaling. The Smad3 can directly bind to the promoter region of collagens and subsequently trigger their production (Isono et al., 2002). Recent study showed that complete deletion of Smad3 in db/db mouse significantly reduced urinary albumin excretion, serum creatinine, mesangial matrix accumulation, and GBM thickness. As shown by immunohistochemistry, the deposition, expression, and transcription of collagen I and IV were significantly inhibited (Xu et al., 2020).

### LRG1 and Abnormal Angiogenesis of DN

DN and diabetic retinopathy are the most common microvascular complications in diabetes. They share similar pathogenesis to some extent. They are both caused by metabolic disorders due to hyperglycemia and characterized by abnormal angiogenesis. Interestingly, LRG1 has been reported to be highly examined in the serum and urine of renal tubular injury and DN. It is recognized as a potential biomarker for kidney disease (Pek et al., 2015; Liu J. J. et al., 2017; Fu et al., 2018; Lee et al., 2018; Hong et al., 2019). Unlike VEGF-A, which is mainly expressed in podocytes, LRG1 is dominantly expressed in glomerular ECs (Haku et al., 2018; Hong et al., 2019). The consideration of several studies indicate that glomerular ECs injury may precede podocyte injury in DN (Neri et al., 1998; Fu et al., 2016). The LRG1 may have a precursory role in initial development of DN via promoting angiogenesis. Indeed, mouse model with upregulated LRG1 expression and increased endothelial tube formation was observed to display characters similar to early stage of DN (Fu et al., 2018). Previous study has demonstrated that the mechanical microenvironment alteration can trigger overexpression of LRG1 in fibro-proliferative disorders (Gao et al., 2019), thus the hyperglycemia may be a potential mechanism for its expression in glomerular ECs of early stage DN. Moreover, it has been demonstrated that glomerular expression of LRG1 is an increased antecedent to VEGF-A in diabetic db/db mice with characters of glomerular hypertrophy and abnormal angiogenesis (Haku et al., 2018). This suggests that LRG1 may have a separate role in angiogenesis and plays a major role in the inception phase of DN, though VEGF-A is involved in abnormal angiogenesis in early stage of DN. In addition, LRG1 has been shown to directly induce VEGF-A expression and promote tumor angiogenesis in colorectal cancer cells (Zhang et al., 2016), while knockdown of LRG1 dramatically reduced VEGF-A expression in mice retina (Wang et al., 2013).

It is reported that the BMP and activin membrane-bound inhibitor (BAMBI)-deficient mice showed to exacerbate proteinuria and induce glomerularendothelial dysfunction by enhancing the ALK1 pathway (Fan et al., 2015). BAMBI is a TGF-β pseudoreceptor that negatively regulates TGF-β signaling. Previous studies revealed that its loss leads to glomerular hypertrophy through accelerating angiogenesis (Guillot et al., 2012, 2013). Opposite to BAMBI, recent study has demonstrated that the LRG1 potentiates ALK1-Smad1/5/8 signaling to promote angiogenesis by interacting with endoglin and TβRII in glomerular ECs of DN mice (Figure 2B). Hong et al. (2019) observed that the phosphorylation of Smad1/5/8 signaling transduction in glomerular ECs exposed to high glucose condition is significantly increased but markedly decreased after deletion of LRG1 gene. In agreement with previous investigation on diabetic retinopathy mice model (Wang et al., 2013), the endoglin and TβRII is found to coimmunoprecipitated with labeled LRG1 in glomerular ECs. Notably, the knockout of LRG1 gene can maintain renal protective effects at a later stage of DN without any unfavorable outcome (Hong et al., 2019). Moreover, several lines of investigations have confirmed that the elevation of LRG1 in plasma and urine of patients is a risk factor for DN progression (Liu J. J. et al., 2017; Hong et al., 2019).

As the kidney functions worsen in the advanced stage of DN, the capillary endothelial cells give rise to capillary loss, tissue hypoxia, and oxidative stress. These would cause the reduction in the local nutrient supply and aggravate renal fibrosis (Cao et al., 2012; Chapal et al., 2013). In addition, previous study showed that hyperactivated endothelial cells and high vascular permeability may also contribute to myofibroblasts (Yamaguchi et al., 2012). However, continuous endothelial activation generally occurred in kidney angiogenesis. Actually, in the study of Haku et al., the fibrosis-related gene expression such as Collagen IV and PAI-1 is observed to be significantly increased in db/db mice at 24 weeks of age (Haku et al., 2018). It is of notice that the glomerular expression of LRG1, VEGF-A, and VEGFR2 is remarkably increased at this DN phase also. At the same time, this mice model exhibits glomerular hypertrophy and abnormal angiogenesis as shown by histological analysis. They proposed that this may be due to the Smad2/3 signaling, which is activated to induce endothelial quiescence as a counterbalance against angiogenesis. As mentioned above, the Smad3 pathway can accelerate fibrotic reaction with an increase in collagen deposition. Moreover, LRG1 is reported to promote lung fibrosis by enhancing Smad2 signaling to activate profibrotic responses in fibroblasts (Honda et al., 2017). Unlike the LRG1-induced Smad1/5/8 signaling, the endoglin is not required for LRG1-induced Smad2 signaling in fibroblasts. In addition, the overexpression of LRG1 is observed to promote epithelial–mesenchymal transition (EMT) by activating MAPK/p38 signaling in thyroid carcinoma cells (Ban et al., 2019). Whereas, EMT has long been implicated in renal tubular fibrosis. Coincidentally, LRG1 also enhances the EMT via HIF-1α activation in colorectal cancer (Zhang et al., 2016).

To sum up, LRG1 might be a potential preemptive therapeutic target of DN, but it needs further studies for better comprehension of clinical implications of LRG1 in DN.

## Other Angiogenic Factors Associated With DN

### Angiopoietins

Angiopoietin is another family of growth factor involved in the progression of DN. Angiopoietin 1 (Ang1) and Ang2 are the two main studied angiopoietins, which bind and signal through a tyrosine kinase receptor referred to as Tie2 (Gnudi, 2016). Ang1 is important in maintaining the stability and permeability of vasculature, whereas the Ang2 functions opposite to Ang1 and promotes vascular wall destabilization (Khoury and Ziyadeh, 2011). Li et al. (2019) revealed that Ang1 could give rise to vascular maturation and stabilization in Matrigel angiogenic model under diabetic conditions. This may be due to Ang1 upregulated expression of junction proteins ZO-1, occlude, and VE-cadherin to enhance tight junction and adhesion junctions of endothelial and pericytes (Li et al., 2019). Recent study revealed that the Tie2 signaling is attenuated by upregulated vascular endothelial protein tyrosine phosphatase in diabetic kidney in mice. While the activation of Tie2 protects the diabetic mice from the development of proteinuria, loss of GFR, and glomerular histopathological changes, this may be due to the activation that initiates the signaling events of PI3K and AKT, which increases eNOS phosphorylation and local NO production (Carota et al., 2019). Consistently, the reduced Ang1/Tie2 signaling leads to increased unstable glomerular capillaries in hyaluronan synthase 2 knockout mouse model (van den Berg et al., 2019; Wang et al., 2020). The potential mechanism may be the loss of hyaluronan that impaired the interaction between endothelial cells and pericytes. The pericytes are cells that wrap around the endothelial cells, which play an important role in stabilizing the blood vessels. The increased Ang2 level contributes to pericyte loss in diabetic mouse (Dewi et al., 2019). The loss of pericytes is previously reported to activate the endothelium and leading to the formation and acellular capillaries (Felcht et al., 2012). Ang2 blocking has been demonstrated to decreasing pericytes loss, increasing endothelial cell junctions, and improving the basal membrane (Holopainen et al., 2012; Dewi et al., 2019).

In total, current studies show the decreased renal Ang1/Ang2 ratio in diabetic kidney disease. This imbalance in favor of Ang2 signaling would result in destabilization of the capillary and increased vascular permeability, which plays a role in the development and progression of microvascular disease related to DN.

### Vasohibin Family

The vasohibin family is a novel protein family angiogenesis regulation, including *vasohibin-1* (VASH1) and its homologous gene, *vasohibin-2* (VASH2) (Sato, 2013). VASH1 is mainly expressed in vascular endothelial cells and acts to terminate angiogenesis; whereas VASH2 is mainly expressed in regions where angiogenesis is active and acts to promote angiogenesis (Ninomiya et al., 2018). Several studies have showed that the renal level of VASH1 was increased in diabetic kidneys, which may be a counterbalance in response to excessive angiogenic milieu with elevation of VEGF-A (Saito et al., 2011; Hinamoto et al., 2014a). This can be further evidenced by the fact of exacerbation of diabetic renal alterations in mice lacking VASH-1 (Hinamoto et al., 2014b). The mouse model is observed with exacerbated glomerular hypertrophy and urinary albumin excretion, as well as increased mesangial matrix. Moreover, the glomerular accumulation of type IV collagen is significantly increased in the GBM and mesangial area. This is in accordance with the exacerbated renal levels of TGF-β and pSmad3, suggesting that VASH1 may also have a role in renal fibrosis in DN. Of notice, the supplementation of exogenous VASH1 can effectively suppress the progression of DN by attenuating the renal dysfunctions mentioned above (Saito et al., 2011). Interestingly, the addition of exogenous VASH1 inhibited the increase of VEGF-A and flik-1, indicating that VASH1 may regulate angiogenesis via VEGF-A signaling. Indeed, VASH1 has demonstrated to inhibit excessive angiogenesis by preventing phosphorylation of VEGFR2 result from high glucose conditions (Nasu et al., 2009).

Recently, Masuda et al. (2018) reported that the endogenous VASH2 mRNA expression was upregulated in renal cortex and mesangial cells in glomeruli. They investigated the pathogenic roles of VASH2 in DN by using VASH2-deficient mice. The increased urine albumin excretion and glomerular volume were significantly improved in VASH2 knockout mice. The immunofluorescence of CD31 showed that the increase of glomerular capillary area was improved as well, which may be due to the deficiency of VASH2 that significantly prevented the enhanced VEGFR2 expression but the elevated VEGF-A levels were not affected (Shen et al., 2012). As mentioned above, VEGF-A is essential for the integrity of glomerular capillary. On the other hand, the VASH2 has been demonstrated to increase the expression of TβRI and promote downstream signaling (Norita et al., 2017). It is also shown to stimulate fibroblast migration and α-SMA expression in cancer cells (Suzuki et al., 2017). Current study in *in vivo* experiment revealed that the deletion of VASH2 in mesangial cells prevented the increase of type IV collagen induced by high glucose (Masuda et al., 2018). Therefore, VASH2 may accelerate mesangial cells to fibroblast-like cells in diabetic kidney disease.

Taken together, current findings suggest that the vasohibin family may be a promising therapeutic target in suppressing both excessive angiogenesis and renal fibrosis in DN.

## Conclusion and Perspective

Abnormal angiogenesis represented by excessive blood vessel formation around glomeruli and increased permeability is associated with glomerular hypertrophy and proteinuria in DN (Osterby and Nyberg, 1987). Current studies have revealed that VEGF-A and LRG1 are important mediators that are involved in the abnormal angiogenesis of DN by promoting glomerular angiogenesis. VEGF-A inhibitory treatments have shown renal protective effects in DN mouse models, but subnormal VEGF-A levels should be avoided. Given the facts that the glomerular LRG1 expression is earlier than VEGF-A, the LRG1 may be a preemptive therapeutic target of DN (Haku et al., 2018). Recent evidence indicates that the genetic deletion of LRG1 can attenuate abnormal angiogenesis and protect renal functions until the later stage of DN in mouse model (Hong et al., 2019). Moreover, it has proved that the double blockade of LRG1 and VEGF-A is better than their individual antibodies in inhibiting the formation of pathogenic vascular tuft in retinopathy mice model (Wang et al., 2013). The decreased renal Ang1/Ang2 ratio would result in the destabilization of the capillary and increased vascular permeability in diabetic kidney. The vasohibin family is possibly involved in mesangial expansion via mediating VEGFR2 signaling. Taken together, these findings strongly suggest that the angiogenic factors, VEGF-A, LRG1, angiopoietins, and vasohibin, are attractive therapeutic targets for patients with DN, but it needs further study to understand their relationships and clinical implications.

## Author Contributions

AZ and YC drafted and finally edited the manuscript. HF, JC, and LH revised the manuscript critically for important intellectual content. All the authors read and approved the final version of the manuscript.

## Conflict of Interest

The authors declare that the research was conducted in the absence of any commercial or financial relationships that could be construed as a potential conflict of interest.
